# Hemoperitoneum due to bleeding from a vein overlying a subserous uterine myoma: a case report

**DOI:** 10.1186/s13256-020-02383-z

**Published:** 2020-05-08

**Authors:** Samaneh Rokhgireh, Abolfazl Mehdizadeh Kashi, Mohammad Kermansaravi, Banafsheh Tajbakhsh, Leila Allahqoli, Ibrahim Alkatout, Sepideh Khodaverdi

**Affiliations:** 1 Endometriosis Research Center, Iran University of Medical Science, Tehran, Iran; 2 Minimally Invasive Surgery Research Center, Iran University of Medical Sciences, Tehran, Iran; 3 Department of Obstetrics and Gynecology, University Hospitals Schleswig-Holstein, University Hospitals Campus Kiel, Kiel School of Gynecological Endoscopy, Arnold-Heller-Strasse 3, Haus 24, 24105 Kiel, Germany; 4 Endometriosis Research Center, Rasoul Akram Hospital, Iran University of Medical Science, P.O. Box 1445613131, Tehran, Iran

**Keywords:** Hemoperitoneum, Subserous myoma, Case report

## Abstract

**Background:**

Fibroids are the most common pelvic tumors in women; serious complications are rare but can be life-threatening.

**Case presentation:**

We present a case report of a 38-year-old Persian woman with acute abdominal pain and a history of uterine fibroids. The patient refused to undergo a laparoscopic myomectomy. Her ultrasound examination revealed free fluid in the abdominal cavity, and her vital signs were indicative of vasogenic shock. A diagnostic laparoscopy was performed to identify and control the source of bleeding: 400 ml of blood and blood clots were removed. Active bleeding was seen from a vein overlying a subserosal myoma. A laparotomic myomectomy was performed, and the patient was discharged 3 days after surgery with no complications.

**Conclusion:**

Surgeons should consider the possibility of this complication in women with acute abdominal pain and a history of uterine leiomyoma.

## Background

Fibroids are the most common pelvic tumors in women [1]; the large majority are asymptomatic and small in size [2]. Symptoms of leiomyoma include abnormal uterine bleeding due to the bulk of the fibroid, as well as reproductive dysfunction [3]. Nevertheless, fibroids may cause serious and acute complications, such as acute urinary retention and renal failure, intraperitoneal hemorrhage, mesenteric vein thrombosis, intestinal gangrene, acute torsion of a subserous leiomyoma, and acute vaginal bleeding [4]. These serious complications are rare but very important for the diagnosis and interdisciplinary differential diagnosis, because they may cause severe morbidity and even mortality. We present a case of intraperitoneal hemorrhage due to a ruptured vein of a subserosal leiomyoma.

## Case presentation

A 38-year-old Persian woman who was a virgin was referred to our emergency ward with right-sided abdominal pain at 11:00 a.m. Her pain had started in the right upper quadrant of the abdomen; its onset was sudden, and it persisted for 2 hours with one fainting episode. The patient’s last menstrual period had been 7 days before admission, and she reported no trauma or previous surgery. Her pulse rate was 110 beats/minute; her systolic blood pressure was 80 mmHg and diastolic blood pressure was undetectable; and her body temperature was 37.5 °C. Her abdominal examination revealed tenderness in the right lower and upper abdomen. She had an obvious tender mass extending to the umbilicus. She had a history of uterine leiomyoma. Laboratory tests and ultrasound were requested. Her hemoglobin, hematocrit, white blood cell count, and platelet count were 11.1 mg/dl, 35%, 13.9 × 10^3^/μL, and 333 × 10^9^/L, respectively. The results of the patient’s echocardiogram (echo) and electrocardiogram, Doppler ultrasound of lower limb arteries, troponin enzyme level, and creatine kinase-MB were normal. The report of an ultrasound examination that had already been done revealed an intramural subserosal leiomyoma measuring 9.5 × 6.5 cm with cystic degeneration in the fundus of the uterus. Both ovaries were normal, and there was free fluid in the abdominal cavity up to Morison’s pouch. Furthermore, a massive fluid collection with internal echoes had been seen in the pelvic cavity, suggestive of a clot. Our differential diagnosis included a ruptured ectopic pregnancy, a hemorrhagic ovarian cyst, and a ruptured ovarian cyst. The patient was admitted to the gynecology ward at 2:00 p.m.

The result of her pregnancy test was negative. At 3:00 p.m., the patient was transferred to the operating room. A diagnostic laparoscopy was performed with the patient under general anesthesia in order to identify and control the source of bleeding. Further reasons for performing the laparoscopy were the patient’s unstable hemodynamic condition and the ultrasound report of a hemoperitoneum.

**Fig. 1:**
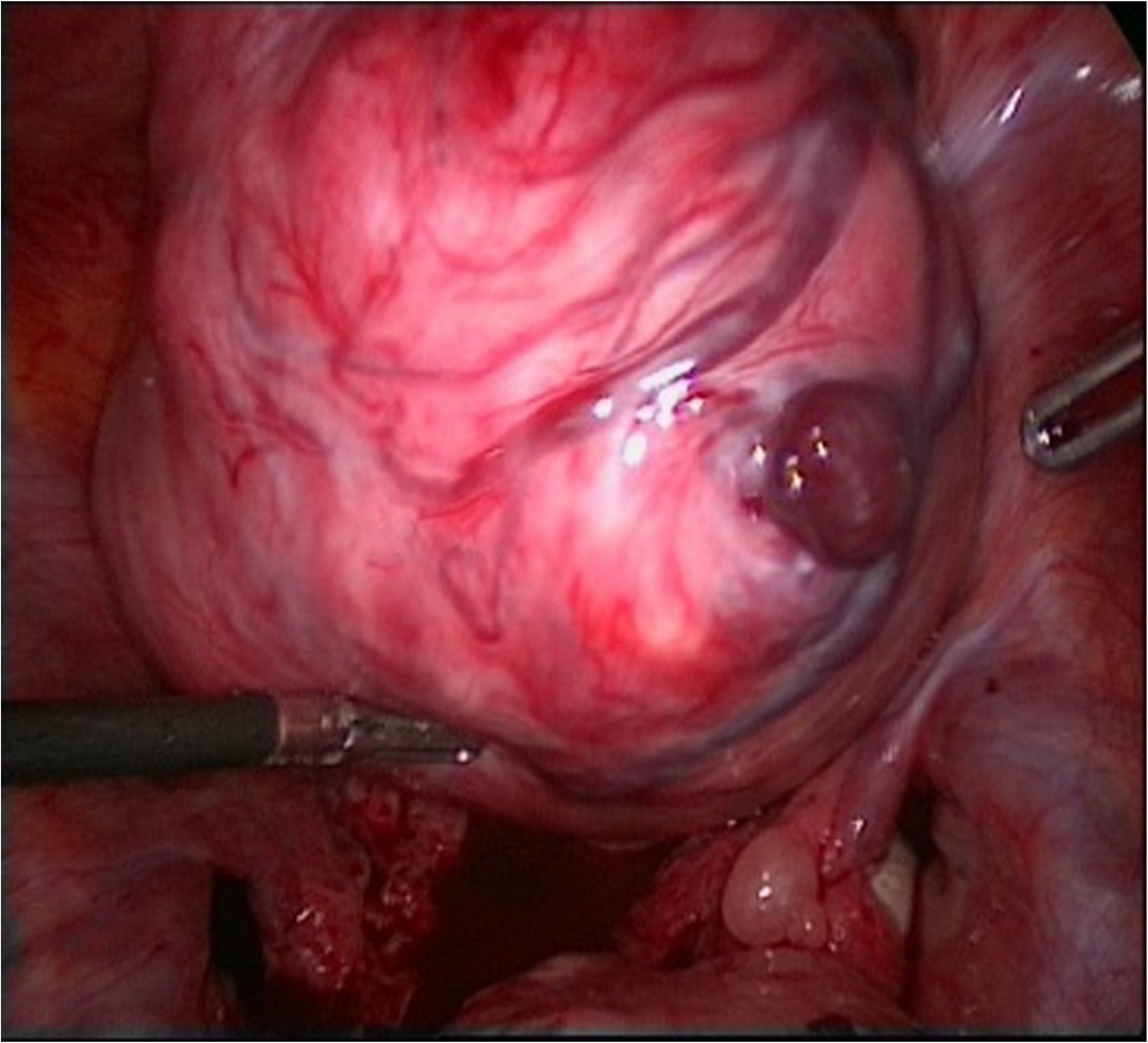
Bleeding vessel on fibroma

A 10-mm trocar was inserted into the umbilicus, and two 5-mm trocars were placed in the right and left lower quadrants. The hemoperitoneum, consisting of 200 ml of fresh blood and 200 ml of clotted blood, was removed. Laparoscopy revealed a large subserosal leiomyoma (10 × 15 cm) located in the fundus of the uterus. The patient’s abdomen and pelvis were examined, and a large subserosal leiomyoma (10 × 15 cm) was found in the fundus of the uterus, with a congested and dilated venous plexus. On the basis of FIGO (International Federation of Gynecology and Obstetrics) classification of uterine fibroids, the fibroid was a pedunculated subserosal fibroid (type 7). A bleeding vessel was seen on the posterior surface of the leiomyoma (Fig. 1). Because the patient refused to undergo a laparoscopic myomectomy, we performed an abdominal myomectomy (laparotomy), and the uterus was preserved during laparotomy. The patient was discharged with no complications on the third day after the operation. At the control investigation performed 2 weeks after surgery, the patient was in normal condition.

## Discussion and conclusions

The uterine leiomyoma is a benign neoplasm. A variety of approaches are used to treat a uterine leiomyoma, such as observation; medical treatment; surgical myomectomy; and, in rare cases, hysterectomy [5]. Intraperitoneal hemorrhage is a rare complication due to bleeding from uterine leiomyoma [6] and is a life-threatening condition [7]. The causes of hemoperitoneum in connection with leiomyoma include a ruptured leiomyoma [8] rupture of a subserosal vein overlying a uterine myoma [9], bleeding from a subserosal artery [10], a lacerated leiomyoma [11], or an avulsed pedunculated leiomyoma [12]. In most cases, bleeding from a uterine leiomyoma has been associated with trauma or torsion of the tumor, but spontaneous rupture of the superficial vessels is extremely rare [13, 14]. The source is mainly venous [8]. Increased abdominal pressure due to hard work, defecation, sports, violent coitus, pregnancy, and menstruation are predisposing factors for rupture of the superficial veins on uterine leiomyoma [9, 15]. Rupture of a blood vessel on a uterine pseudotumor leading to an isolated hemoperitoneum in the immediate postpartum period has also been reported [16, 17].

A specific preoperative diagnosis can rarely be established in these cases. In one study, the correct diagnosis was made preoperatively in a mere 7.8% of cases [18]. Computed tomography and ultrasound are able to show the hemoperitoneum but usually do not reveal the source of the bleeding [19]. The preoperative diagnosis is commonly an acute abdomen or a hemoperitoneum of unknown origin [8]. In these cases, timely diagnosis and emergent surgery, extending from ligation of the bleeding vessel to myomectomy or hysterectomy, can prevent catastrophic consequences [15]. The decision depends on the patient’s age and her desire to preserve fertility [20]. Previously reported cases of hemorrhage associated with uterine leiomyoma were successfully managed with emergency laparotomy, which proved to be a lifesaving measure [15, 20]. In our patient’s case, laparoscopy was performed for the diagnosis of the source of the bleeding, the uterus was preserved during laparotomy, and the uterine leiomyoma was treated by surgical excision.

Despite its rarity, a bleeding leiomyomatous vessel should be included in the differential diagnosis of a hemoperitoneum of ambiguous origin. Rapid diagnosis and management are essential in this potentially life-threatening condition. Surgeons should consider the possibility of this complication in women with acute abdominal pain and a history of uterine leiomyoma in order to prevent severe morbidity or even mortality. When a patient undergoes conservative management of large asymptomatic subserosal fibromas, she should be informed of this rare complication.

## Data Availability

Clinical data and complementary examinations are available from the corresponding author on reasonable request.

## Abbreviation

FIGO: International Federation of Gynecology and Obstetrics
